# Women’s health across the reproductive lifespan in women with germline *TP53* pathogenic variants: a clinical perspective

**DOI:** 10.1093/oncolo/oyag212

**Published:** 2026-06-11

**Authors:** Naama Halpern, Rinat Bernstein-Molho, Yehudit Peerless, Iris Kventsel, Zehavit Frenkel, Yossi Mizrachi, Hagit Shani, Myriam Safrai

**Affiliations:** The Jusidman Cancer Center, Sheba Medical Center, Ramat Gan 5265601, Israel; Gray Faculty of Medical and Health Sciences, School of Medicine, Tel-Aviv University, Tel Aviv-Yafo, 6997801, Israel; The Jusidman Cancer Center, Sheba Medical Center, Ramat Gan 5265601, Israel; Gray Faculty of Medical and Health Sciences, School of Medicine, Tel-Aviv University, Tel Aviv-Yafo, 6997801, Israel; The Suzanne Levy-Gertner Oncogenetics Unit, Danek Gertner Institute of Human Genetics, Sheba Medical Center, Ramat Gan 5265601, Israel; The Jusidman Cancer Center, Sheba Medical Center, Ramat Gan 5265601, Israel; Department of Pediatric Hemato-Oncology, Edmond and Lilly Safra Children’s Hospital and Cancer Research Center, Sheba Medical Center, Ramat Gan 5265601, Israel; Department of Pediatric Hemato-Oncology, Edmond and Lilly Safra Children’s Hospital and Cancer Research Center, Sheba Medical Center, Ramat Gan 5265601, Israel; Gray Faculty of Medical and Health Sciences, School of Medicine, Tel-Aviv University, Tel Aviv-Yafo, 6997801, Israel; Fertility Unit, Edith Wolfson Medical Centre, and Gray Faculty of Medical Sciences, Tel-Aviv University, Tel Aviv-Yafo 5822012, Israel; Preimplantation Genetic Diagnosis Center, Danek Gertner Institute of Human Genetics, Sheba Medical Center, Ramat Gan 6997801, Israel; Gray Faculty of Medical and Health Sciences, School of Medicine, Tel-Aviv University, Tel Aviv-Yafo, 6997801, Israel; Fertility Unit, Edith Wolfson Medical Centre, and Gray Faculty of Medical Sciences, Tel-Aviv University, Tel Aviv-Yafo 5822012, Israel

**Keywords:** *TP53* pathogenic sequence variants, Li-Fraumeni syndrome, reproductive health, fertility preservation, hormonal therapy, women’s health

## Abstract

Li-Fraumeni syndrome, caused by germline *TP53* pathogenic sequence variants (PSVs), is among the most penetrant hereditary cancer syndromes. With broader genetic testing and improved survivorship, more women with *TP53* PSVs now seek reproductive and hormonal guidance throughout their lifespan. Yet, no evidence-based framework currently exists to support women’s health care in this population. In this review, we searched the available literature and found an absence of data regarding contraception, fertility preservation, pregnancy, and menopause, resulting in inconsistent counseling and both under- and overtreatment. Drawing on analogies from other high-risk cancer syndromes and our multidisciplinary clinical experience, we present interim suggestions centered on shared decision-making, individualized hormonal management, and avoiding unnecessary interventions. These findings underscore the need for prospective studies and international registries to establish evidence-based and equitable women’s health care for women with *TP53* PSVs.

Implications for practice Women with germline *TP53* pathogenic sequence variants associated with Li-Fraumeni syndrome increasingly seek guidance on reproductive and hormonal health, yet evidence-based recommendations remain limited. This review highlights key knowledge gaps and proposes interim, clinically pragmatic approaches to contraception, fertility preservation, pregnancy, and menopause care. Emphasizing shared decision-making and individualized risk assessment, these considerations may assist clinicians in providing consistent counseling while avoiding unnecessary interventions. The manuscript also underscores the urgent need for prospective studies and international registries to establish evidence-based guidelines for women’s health management in this high-risk population.

## Introduction

Li-Fraumeni syndrome (LFS), driven by germline pathogenic sequence variants (PSVs) in the tumor suppressor gene *TP53* (OMIM #151623), is one of the most penetrant hereditary cancer predisposition syndromes.^1^ The prevalence of *TP53* PSVs is estimated at approximately 1:3000 to 1:10 000 in unselected populations.^2^ Individuals with *TP53* PSVs face high lifetime risks of diverse malignancies, especially premenopausal breast cancer, soft-tissue sarcoma, central nervous system tumors, and adrenocortical carcinoma.^3^^,^^4^ The lifetime cancer risk approaches 90%-100% in women and is substantially elevated in men.^5^^,^^6^ Among women with *TP53* PSVs, the risk of developing any cancer type by age 30 is 40%-50%, with about a 7% risk of developing breast cancer before the age of 30 years.^5^^,^^6^ Breast cancer among women with *TP53* PSVs is more frequently characterized by human epidermal growth factor receptor 2 (HER2) amplification, with some tumors exhibiting triple-positive subtypes.^4^^,^^7–9^ These triple-positive tumors express estrogen and progesterone receptors (ER, PR) in addition to HER2, reflecting their hormone-dependent biology. Recent data also suggest a possible association between Li-Fraumeni syndrome and ovarian cancer risk. In a large cohort analysis, Fortuno et al reported an increased odds ratio (∼11) for ovarian cancer among women with *TP53* PSVs.^6^ However, these findings remain hypothesis-generating, as key limitations include lack of histologic subtype data and the potential contribution of clonal hematopoiesis (CHIP), which may confound germline interpretation. These uncertainties currently preclude changes to clinical management but warrant further investigation.

Historically, most women with *TP53* PSVs who came to medical attention had a personal history of cancer or were members of families with multiple early-onset tumors. Their care largely centered on surveillance, secondary cancer prevention, and oncologic treatment.^10^ Over the past decade, 3 developments have transformed the clinical landscape: First, the widespread adoption of next-generation sequencing has enabled the identification of individuals with *TP53* PSVs outside “classic” LFS pedigrees, often healthy women without striking family histories, with an attenuated phenotype.^11–13^ Second, structured surveillance protocols, including whole-body MRI (WBMRI) and targeted organ screening, have improved early detection and survival.^14–16^ Screening recommendations continue to evolve, with recent updates reinforcing the role of WBMRI-based surveillance protocols and emphasizing individualized risk-adapted management.^17^^,^^18^ Third, advances in oncology care have extended life expectancy, resulting in a growing number of women with *TP53* PSVs living into their reproductive years and beyond.

As a result, an increasing number of women with *TP53* PSVs is raising questions about contraception, family planning, pregnancy, and menopause. Yet no clinical guidelines or consensus statements address these issues. In practice, management varies widely; some women are denied basic options such as contraception, while others undergo unnecessary interventions such as prophylactic surgery, highlighting the need for evidence-based guidance.

This review focuses on health issues that most commonly arise in the reproductive lifespan in the clinical care of women with *TP53* PSVs. These topics were selected based on recurring concerns raised by unaffected women with *TP53* PSVs and cancer survivors in our LFS dedicated high-risk hereditary cancer clinic, as well as gaps identified in the existing literature. We summarize key challenges from contraception to menopause, describe our clinical approach as a referral center in the absence of established recommendations, propose interim guidance (Table 1), and call for international collaboration to develop evidence-based practices.

**Table 1.** Key clinical considerations across the reproductive lifespan in women with germline *TP53* pathogenic sequence variants.

Domain	Clinical question	Evidence/analogies	Suggestion
**Contraception**	Hormonal contraception safety given hormone-related cancer risk	No *TP53*-specific data; *BRCA1/2* show minimal risk increase with long-term use	Shared decision; prefer nonhormonal (copper IUD, barrier); short-term hormonal acceptable with counseling & surveillance
**Fertility preservation**	Timing/safety of ovarian stimulation	No *TP53*-specific guidance; ∼50% develop malignancy by 30; estrogen minimized with tailored protocols	Early reproductive counseling & referral; use low-estrogen protocols
**PGT-M/oocyte donation**	Preventing variant transmission; hormone safety	PGT-M feasible; estrogen exposure can be minimized	Offer IVF with PGT-M or oocyte donation; use low-estrogen protocols
**Pregnancy**	Cancer risk & surveillance feasibility	No evidence of increased risk; US safe for surveillance. Complete MRI pre-pregnancy if feasible	Do not discourage pregnancy; confirm disease-free status; maintain US surveillance, MRI if necessary without gadolinium
**Menopause/HRT**	Safety of hormone therapy	No *TP53*-specific data; *BRCA1/2* show no added risk; HRT remains first-line	No contraindication; discuss hormonal/nonhormonal options; use lowest effective dose, reassess regularly
**Risk-reducing salpingo-oophorectomy**	Prophylactic surgery without evidence	Ovarian cancer not core LFS tumor; no data support surgery	Avoid unnecessary surgery; emphasize conservative management
**Psychosocial/multidisciplinary care**	Emotional burden & uncertainty	Multidisciplinary counseling improves coping & understanding	Provide integrated counseling, psychosocial support; encourage registry participation

Abbreviations: IUD—intrauterine device; PGT-M—preimplantation genetic testing for monogenic disorders; IVF—in vitro fertilization; HRT—hormone replacement therapy; LFS—Li-Fraumeni syndrome.

## Contraceptive choices

### Question

Cancer risk in individuals with *TP53* PSVs often overlaps with the reproductive years, when contraception is commonly needed. This raises the question of whether hormonal contraception should be avoided or used cautiously in this population, given the theoretical concern that hormone exposure might influence malignancy risk.

### Current evidence and analogies

No published studies have specifically addressed the safety of hormonal contraceptives in women with *TP53* PSVs. In the general population, hormonal contraceptives are associated with a slight increase in breast cancer risk, although the absolute excess risk remains minimal.^19^ Among individuals with *BRCA1/2* PSVs, some studies suggest an elevated risk associated with hormonal contraceptive use, particularly with prolonged use (>5 years) and mainly in women with a *BRCA1* PSV.^20–22^ However, the evidence remains inconsistent, with other studies reporting no significant association with breast cancer risk. In contrast, oral contraceptive use has been associated with a reduced risk of ovarian cancer in this population.^23^^,^^24^ Consequently, oral combined or progestin-only contraceptives are not contraindicated for women with *BRCA1/2* PSVs.^25^^,^^26^ Data specific to patients with *TP53* PSVs remain extremely limited; available evidence suggests that oral contraceptive use does not significantly affect breast cancer risk in this population, although these findings are based on limited data and should be interpreted cautiously.^27^

### Clinical observations

Counseling regarding contraception in patients with *TP53* PSVs is often overly cautious, and some women are denied hormonal methods despite the absence of direct evidence demonstrating harm. We suggest that counseling follow a shared decision-making model, in which the lack of syndrome-specific evidence and the potential, albeit unquantified, risks are openly discussed. For long-term contraception, nonhormonal methods such as a copper intrauterine device (IUD) or barrier methods are generally preferred. However, when shorter-term contraception is needed, hormonal options may also be considered after individualized counseling. Importantly, hormonal contraceptives are frequently prescribed not only for contraception but also for broader women’s health indications, including acne, menorrhagia, or Polyendocrine Metabolic Ovarian Syndrome (PMOS), which may significantly affect quality of life if left untreated.

### Interim suggestion

Use shared decision-making, clearly communicating uncertainty. Nonhormonal methods may be preferred for long-term contraception. However, hormonal contraception is not contraindicated and may be considered after discussion of theoretical risks, with periodic reassessment based on individual risk profile and clinical needs.

## Fertility preservation

### Question

Given the high lifetime malignancy risk and frequent need for urgent fertility preservation, should unaffected women with *TP53* PSVs receive proactive fertility preservation counseling?

### Current evidence and analogies

No specific guidelines exist. Once malignancy is diagnosed, fertility preservation is typically limited to 1 or 2 emergency stimulation cycles before therapy.^28^^,^^29^ In women under 35, about 8-10 mature oocytes are needed for 1 live birth; at ages 35-40, 15-20; and over 40, more than 20.^30^^,^^31^ For carriers pursuing preimplantation genetic testing for monogenic disorders (PGT-M), roughly half of embryos will test positive, effectively doubling oocyte requirements.

To allow effective fertility preservation process, the Society of Gynecologic Oncology and the American Society for Reproductive Medicine recommend early referral of patients with *BRCA1/2* PSVs in their late 20s to early 30s to reproductive endocrinologists, so that they can be informed of the availability of fertility preservation and the potential for PGT-M.^32^ Although women with *TP53* PSVs do not face prophylactic salpingo-oophorectomy, their younger median age at cancer onset and the option of PGT-M may support similarly early reproductive counseling.

Recent population-based evidence evaluating cancer incidence following medically assisted reproduction did not demonstrate an increase in overall cancer incidence, although small increases in selected cancer types were reported.^33^ Concern regarding ovarian stimulation in women with *TP53* PSVs centers on transient supraphysiologic estradiol exposure. Direct *TP53*-specific safety data are lacking; however, available evidence from breast cancer patients undergoing fertility preservation has not shown worse oncologic outcomes, and evidence from patients with *BRCA1/2* PSVs has not demonstrated an increased breast cancer risk after assisted reproductive technology.^34–37^ To minimize supraphysiologic estrogen exposure, protocols incorporating aromatase inhibitors such as letrozole or selective estrogen receptor modulators such as tamoxifen can be used with the addition of a gonadotropin-releasing hormone (GnRH) trigger.^38–41^ This approach, endorsed by the Society of Gynecologic Oncology and the American Society for Reproductive Medicine, represents the standard of care for hormone-sensitive breast cancer patients and may provide a safer hormonal profile for women with *TP53* PSVs without reducing treatment efficacy.^32^^,^^42^

A substantial proportion of patients with *TP53* PSVs who manifest early-onset disease are diagnosed in early childhood, with many developing malignancies before puberty.^43^ In these patients, oocyte cryopreservation is not feasible, and ovarian tissue cryopreservation (OTC) represents the only available fertility preservation option. OTC is a brief surgical procedure that does not delay oncologic treatment and may be performed even after chemotherapy initiation. Therefore, it is recommended only in the setting of a concrete risk of treatment-related infertility.^44^ Routine proactive fertility preservation counseling at the time of *TP53* PSV detection in prepubertal girls is not indicated.

### Clinical observations

Beyond the clinical considerations, fertility preservation may impose a tremendous psychological burden for women carrying *TP53* PSVs, often beginning in their early reproductive years. The awareness of potential cancer risk and the prospect of passing on the pathogenic variant through natural conception can create profound anxiety and decisional stress. Although fertility preservation and PGT-M options exist, these discussions are not always initiated early within surveillance programs. Moreover, some young adult women with *TP53* PSVs remain under the care of pediatric oncologists or genetic clinics less familiar with reproductive health management, potentially leading to missed opportunities for anticipatory counseling and timely referral to reproductive endocrinologists.

### Interim suggestion

Reproductive decision-making for women with *TP53* PSVs is medically and emotionally complex, and the likelihood of requiring fertility preservation is high. Referral to a reproductive endocrinologist is recommended from late adolescence, when oocyte cryopreservation becomes feasible. Counseling should address both elective and urgent options, including PGT-M. If ovarian stimulation is undertaken, protocols that minimize estrogen exposure are preferred. In prepubertal patients, fertility preservation should be considered only in the setting of a cancer diagnosis.

## Preimplantation genetic testing and oocyte donation

### Question

Which reproductive options are available to individuals with *TP53* PSVs to prevent the transmission of variants, and what are the roles of PGT-M and oocyte donation?

### Current evidence and analogies

The safety considerations surrounding ovarian stimulation have been discussed above. When in vitro fertilization (IVF) is undertaken for reproductive purposes in patients with *TP53* PSVs, its role extends beyond fertility treatment to include the option of PGT-M.

PGT-M enables selection of embryos free of the familial *TP53* PSV and has been widely implemented in other hereditary cancer syndromes, including hereditary breast and ovarian cancer, Lynch syndrome, and familial adenomatous polyposis.^45^ Although data specific to individuals with *TP53* PSVs remain limited to isolated reports,^46^ the syndrome fulfills accepted criteria for PGT-M, including high penetrance, severe phenotype, and frequent early-onset malignancies.^47^ Notably, recent data suggesting transmission ratio distortion in *TP53* PSVs inheritance raises the possibility that the likelihood of transmitting the pathogenic variant may exceed the expected 50%,^48^ further strengthening the rationale for offering PGT-M. However, systematic data regarding uptake, reproductive outcomes, oncologic safety, and psychosocial impact are lacking.

Oocyte donation represents an established reproductive option for women who wish to eliminate transmission risk without undergoing PGT-M, or in cases of diminished ovarian reserve, including following chemotherapy. Professional societies endorse gamete donation for individuals carrying significant hereditary cancer syndromes.^49–51^ For some women, oocyte donation provides a pathway to motherhood while avoiding both genetic transmission and repeated ovarian stimulation.

### Clinical observations

In our high-risk clinic, PGT-M is routinely offered to both women and men with *TP53* PSVs and demonstrates high acceptance. Over the past decade, 10 children have been born following PGT-M in families under surveillance. For many couples, PGT-M alleviates the ethical and emotional burden associated with prenatal diagnosis and potential termination.

### Interim suggestion

Given the complex ethical and emotional considerations surrounding reproductive decision-making in patients with *TP53* PSVs, counseling should be provided within a multidisciplinary framework that includes expertise in reproductive endocrinology, oncology, and genetics. Counseling should include discussion of PGT-M alongside oocyte donation and should be tailored to individual medical, ethical, financial, and psychosocial factors. Systematic collection and reporting of reproductive outcomes are essential to inform future evidence-based counseling and care.

## Pregnancy and reproductive outcomes

### Question

Does pregnancy increase cancer risk or alter outcomes in women with *TP53* PSVs, and how should surveillance be modified?

### Current evidence and analogies

No prospective data have specifically evaluated whether pregnancy increases cancer risk or worsens oncologic outcomes for women with *TP53* PSVs. Physiologic pregnancy is characterized by elevated estrogen, progesterone, growth factors, and immune modulation, all of which could theoretically influence tumor initiation or progression; however, this has not been demonstrated. Data from breast cancer survivors in the general population, including women with estrogen receptor–positive disease, do not show adverse effects of subsequent pregnancy on recurrence or survival.^52^^,^^53^ In patients with *TP53* PSVs, retrospective data suggest that older age at first pregnancy may be associated with increased breast cancer risk, although these findings require cautious interpretation and further validation.^27^

Guidance on breast surveillance during pregnancy and lactation is largely extrapolated from recommendations for the general population and for women with *BRCA1/2* PSVs; during pregnancy, surveillance relies primarily on clinical breast examination and breast ultrasound.^54^^,^^55^ Mammography may be performed when clinically indicated, whereas routine contrast-enhanced breast MRI is generally deferred until postpartum because gadolinium is usually avoided during pregnancy.^54^ MRI without gadolinium may be performed during pregnancy when clinically indicated for systemic workup.^56^ During lactation, MRI may be resumed according to the patient’s high-risk surveillance schedule.^55^ WBMRI may also be performed for systemic workup during pregnancy in breast cancer patients.

Surrogacy laws and access vary globally. It is an accepted option when pregnancy is contraindicated or poses health risks. The Society of Gynecologic Oncology and the American Society for Reproductive Medicine endorse surrogacy for women with hereditary gynecologic cancers after hysterectomy.^32^ Although there is no formal indication for surrogacy for women with *TP53* PSVs, it may be discussed in selected cases in which prior cancer history, treatment-related morbidity, or individualized medical concerns make pregnancy inadvisable.

### Clinical observations

In our high-risk hereditary cancer clinic, women carrying *TP53* PSVs often face complex challenges regarding pregnancy, including balancing cancer risk, surveillance demands, and the possibility of transmitting the variant.

### Interim suggestion

Pregnancy should not be routinely discouraged solely on the basis of a germline *TP53* PSV. Provide preconception counseling with maternal-fetal medicine and oncology teams to review potential risks and establish an individualized surveillance plan. Confirm disease-free status before conception and maintain close monitoring during pregnancy, postpartum, and lactation per LFS surveillance protocols. For breast cancer screening, we recommend performing clinical breast examination and breast ultrasound (US) every 3 months during pregnancy and lactation, as well as breast MRI after birth.

## Hormone therapy in postmenopausal women

### Question

Is hormone replacement therapy (HRT) appropriate for managing menopause in women with *TP53* PSVs?

### Current evidence and analogies

No study specifically addresses HRT safety in women with *TP53* PSVs. With the growing recognition of attenuated forms of LFS, earlier cancer detection, and improved survival, we expect more women with *TP53* PSVs to reach natural menopause. The absence of data specific to women with *TP53* PSVs may leave these patients vulnerable to undertreatment, potentially compromising their quality and longevity of life without an evidence-based rationale.^57^

HRT is the most effective therapy for vasomotor and genitourinary symptoms and supports bone and cardiovascular health.^58^ According to the 2022 North American Menopause Society (NAMS) position statement, it has a favorable benefit-risk profile for symptomatic women younger than 60 years or within 10 years of menopause onset, whereas initiation after age 60 or more than 10 years from menopause is less favorable because of higher absolute cardiometabolic and neurologic risks.^59^ Importantly, these recommendations do not specifically account for *TP53*-related hereditary cancer risk.

The formulation, regimen, and timing of HRT are also clinically relevant. In women with an intact uterus, progestogen is required for endometrial protection, whereas estrogen-only therapy used in women without a uterus appears to have a more favorable breast cancer risk profile.^60^ By contrast, combined estrogen–progestogen therapy is associated with increased breast cancer risk,^61–63^ which varies according to progestogen type, regimen, and duration.^64^^,^^65^ Combined regimens are generally associated with a greater risk than sequential approaches. Micronized progesterone, dydrogesterone, or tissue-selective options such as conjugated estrogens with bazedoxifene may offer alternative strategies in selected patients, although long-term data remain limited.^61^^,^^64^ This distinction is particularly relevant for populations with elevated baseline breast cancer susceptibility, including patients with *TP53* PSVs. Data from women with *BRCA1/2* PSVs may offer partial analogies but should be interpreted cautiously. Short-term HRT following risk-reducing salpingo-oophorectomy between ages 35 and 45 appears relatively reassuring in women with *BRCA1/2* PSVs; however, Michaelson-Cohen et al reported a 3-fold increased breast cancer risk in carriers older than 45 years, suggesting that timing of exposure relative to natural menopause may be particularly relevant in hereditary cancer populations.^66^

For women who cannot or prefer to avoid systemic hormones, nonhormonal therapies, including selective serotonin reuptake inhibitors (SSRIs), serotonin–norepinephrine reuptake inhibitors (SNRIs), gabapentin, and neurokinin-targeted agents such as fezolinetant or the dual NK1/NK3 antagonist elinzanetant may provide clinically meaningful symptom relief.^67–69^ For isolated genitourinary symptoms, low-dose vaginal estrogen, vaginal dehydroepiandrosterone, or oral ospemifene may be considered, with minimal systemic exposure relative to systemic HRT.

### Clinical observations

Some women are denied HRT despite menopausal symptoms impacting their quality of life, while others receive HRT without structured monitoring.

### Interim suggestion

Manage HRT on a case-by-case basis, considering symptom type, severity, available treatments, patient preference, and syndrome phenotype. Although no contraindication exists for HRT in women with *TP53* PSVs, patients should be informed about nonhormonal options offering promising alternatives, especially for vasomotor symptom relief. If HRT is chosen, use the lowest effective dose, maintain regular surveillance, and periodically reassess the risk–benefit balance.

## Special populations: gender-diverse individuals

Recent LFS surveillance updates also highlight the need to consider gender-diverse individuals, including transgender and nonbinary patients, whose care may involve gender-affirming hormone therapy or surgery.^17^ In individuals with *TP53* PSVs, these decisions should be individualized and addressed within a multidisciplinary framework that includes genetics, oncology, endocrinology, reproductive medicine, and mental health expertise. Because *TP53*-specific data regarding gender-affirming hormone exposure are lacking, counseling should explicitly address uncertainty, cancer surveillance implications, and the need to adapt breast/chest screening according to anatomy, prior surgery, hormone exposure, and individual cancer risk.

## Overtreatment and unwarranted interventions

In this context, unnecessary intervention refers to risk-reducing procedures performed in the absence of evidence-based indication or guideline support. This distinction is important in women with *TP53* PSVs, because risk-reducing mastectomy is an established option given the high lifetime risk of early-onset breast cancer and should not be considered overtreatment when appropriately indicated.

By contrast, current evidence does not support routine risk-reducing salpingo-oophorectomy (RRSO) solely on the basis of a germline *TP53* PSV. Although emerging data suggest a possible increased ovarian cancer risk in women with *TP53* PSVs, these findings remain preliminary and hypothesis-generating^6^ and do not currently support routine RRSO. Unwarranted premenopausal RRSO causes immediate menopause and infertility, and may lead to sexual dysfunction, bone loss, cardiometabolic consequences, and reduced quality of life. Counseling should distinguish evidence-supported risk-reducing strategies from fear-driven or non–guideline-supported interventions.^70^^,^^71^

## Conclusion

Women with germline *TP53* PSVs increasingly seek comprehensive reproductive and hormonal guidance through various phases of their lives. Despite this growing clinical need, evidence-based recommendations for contraception, fertility preservation, pregnancy, and menopause management remain absent in this population. In practice, both under- and overtreatment are observed, often resulting in avoidable compromises to health and quality of life.

Until prospective data and formal guidelines are available, care should emphasize multidisciplinary shared decision-making, transparent communication about uncertainty, and cautious individualized management. Rare conditions such as LFS should ideally be managed by clinicians experienced in caring for this specific population. Establishing international registries, prospective cohorts, and collaborative clinical studies is essential to generate the evidence needed to inform safe, equitable, and evidence-based care for women with *TP53* PSVs.

## Data Availability

All relevant data are contained within the publication itself.
